# Obstetric racism and perceived quality of maternity care in Canada: Voices of Black women

**DOI:** 10.1177/17455057231199651

**Published:** 2023-09-29

**Authors:** Priscilla N Boakye, Nadia Prendergast, Bahareh Bandari, Eugenia Anane Brown, Awura-ama Odutayo, Sharon Salami

**Affiliations:** 1 Toronto Metropolitan University, Toronto, ON, Canada; 2 University of Toronto, Toronto, ON, Canada

**Keywords:** Black women, Obstetric racism, Maternal health, Quality of care, Canada

## Abstract

**Background:**

Obstetric racism in healthcare encounters impact on access to quality maternal healthcare for Black childbearing women yet remains underexplored in Canada. Understanding the experiences of Black Canadian women is critical to inform policy and create targeted interventions to address obstetric racism and advanced maternal health equity.

**Objective:**

The aim of this study was to explore the experiences of obstetric racism and its influence on perceived quality of maternity care among Black women in Toronto, Canada.

**Design:**

Qualitative research was conducted using a critical qualitative inquiry approach.

**Methods:**

We conducted a semi-structured interviews with 24 Black women who were pregnant and/or have given birth in the last 3 years. The interviews explored their experiences seeking care during pregnancy/childbirth and perceived quality of care.

**Results:**

Two themes were generated through the process of thematic analysis: (1) Manifestations and Impacts of Obstetric Racism and (2) Strategies for Addressing Obstetric Racism. Narratives of being dismissed, objectified, dehumanized, trauma and paternalism were reflected in the accounts of the participants. These experiences undermined the quality of care, hindered therapeutic relationships and contributed to mistrust.

**Conclusion:**

Black women understood the nature and impact of obstetric racism as it relates to the quality of maternal health care, their safety, and well-being. Participants recommended the need for anti-Black racism training specific to caring of Black childbearing women and increasing Black healthcare provider representation in perinatal settings as strategies to address obstetric racism. Investment in Black maternal health research is urgently needed to generate meaningful evidence to inform policy and interventions to advanced maternal health equity.

## Plain Language Summary

Obstetric racism may affect access to quality maternal healthcare and contribute to poorer health outcomes for Black women. We asked Black women in Toronto to share their experiences receiving care during pregnancy or childbirth from healthcare providers and how they felt about the care. Black women reported that their care was dehumanizing and lacking in quality. Training and increasing Black healthcare provider representation were identified as strategies to prevent obstetric racism and improve the quality of maternal healthcare.

## Introduction

Adverse Black maternal health outcomes have worsened in developed countries, such as the United States and the United Kingdom.^1–3^ The risk of adverse maternal health outcomes among Black women in these countries is approximately three to four times higher when compared with other groups.^2,4^ In Canada, unlike the United States and the United Kingdom, few health agencies collect and report racial data and statistics on women’s health, which reflects a ‘colour-blind’ attitude towards health.^5^ While there are no many studies examining disparities in maternal health outcomes in Canada, a recent study indicates significant disparities, with a preterm birth rate of 8.9% among Black women compared to 5.9% among White women.^6^ Public health and epidemiology researchers have established a positive correlation between adverse maternal health outcomes among Black women and systemic racism and other social inequalities. Racism as a determinant of health, along with the intersection of class, gender, and access to health services, adversely affects Black women’s health outcomes.^7–11^

Disparities in Black maternal health outcomes have been explained by the theories of epigenetics and the disruptive effects of structural racism including inflammatory response and pivotal allostatic imbalance triggering conditions such as preterm labour, gestational diabetes and preeclampsia.^10,12^ Beyond these theories of epigenetics, other researchers have reported that obstetric racism-related barriers to accessing quality prenatal and perinatal care in healthcare institutions may increase the risk of adverse maternal and newborn outcomes for Black women.

Obstetric racism comprises of ‘beliefs and practices that harm the reproducing Black body’ (p. 58) and manifested as forms of violence and abuse experienced by Black women when accessing maternal healthcare services.^13^ The term was coined by Davis^14^ to advance a more theoretical understanding of maternal healthcare encounters of Black women which she argues lies at the intersection of obstetric violence and medical racism. Obstetric violence is defined as acts of commission or omission perpetrated against women by health providers and medical institutions intentionally or unintentionally, resulting in the domination of women’s bodies, control over their reproduction, dehumanized care, medicalization and pathologizing natural reproductive processes, and loss of autonomy and the ability to have control over their decisions, all of which contributes to negatively affecting their well-being and quality of life.^15^

Davis argues that while obstetric violence provides a frame of analysis for deciphering the abuse that occurs in maternal healthcare practice, it does not adequately account for ways racism influences the care and interaction of Black women with healthcare professionals.^14^ Therefore, to understand obstetric racism, it is important to situate Black women’s experiences within the context of medical racism, which refers to ways race and racism interferes with healthcare providers’ perspectives regarding the diagnosis and treatment of certain groups of people, which in turn contributes to adverse health outcomes.^14,16^

Obstetric racism is influenced and shaped by the history of medical experimentation of enslaved women.^14,17^ The manifestation of obstetric racism consists of presumptions, discriminatory beliefs and practices against pregnant and birthing Black women.^13,14,17^ Historically, Black women became victims of experimental gynaecological surgeries without anaesthesia due to the racist belief that Black people have a high pain tolerance.^7,14,17–19^ These acts of injustice fuelled by history of reproductive and medical exploitation of enslaved women gave birth to modern-day obstetric practices^18–20^ and set the precedence for how Black childbearing women are expected to be treated within the healthcare system and by healthcare providers.^13,14^

Obstetric racism is experience in subtle and overt ways including neglect, dismissal of concerns, disrespect, performing non-consensual procedures, and surrogate decision-making.^14^ Other experiences of obstetric racism such as implicit bias, discrimination, stereotype, and microaggressions within healthcare institutions hinder Black women’s access to appropriate, safe, and quality reproductive and maternal healthcare, all of which significantly impacts on maternal health outcomes.^21–23^ In addition, feelings of being unheard and being denied of pain medication were commonly shared by Black women in the United States.^24–26^ These experiences hinder therapeutic relationships resulting in inadequate care, unmet care needs, mistrust, and poor health outcomes.^10,11,23,24,26–28^ There is also evidence linking the experience of abuse and disrespect in perinatal care to increase risk of perinatal mood disorders and psychosocial distress.^29^

Despite the repeatedly documented experiences of obstetric racism elsewhere, no studies in Canada have explored Black women’s perception and understanding of obstetric racism when accessing care during pregnancy and childbirth. Researchers from the United States have found that Black women, irrespective of their educational and socioeconomic status and ability to afford healthcare, still experience obstetric racism when accessing perinatal care.^8,30^ While these studies from the United States share valuable insight for understanding the experiences of Black Canadian women, differences in the social and healthcare contexts between Canada and the United States limits the application of the findings. This study aims to explore Black Canadian women’s experiences of obstetric racism and its influence on perceived quality of maternity care during pregnancy and childbirth.

## Methodology

We conducted a qualitative study with 24 Black women who reside in the Greater Toronto Area (GTA). A generic qualitative design allows researchers the flexibility to draw on appropriate methods and techniques from different qualitative designs to answer a research question.^31^ We adopted a critical qualitative inquiry approach to centre the voices of our participants and to expose how systemic inequality and discrimination manifest in participants’ healthcare encounters.^32^ This approach to inquiry allow researchers to not only interpret the experiences of participants but also present ‘life documents that speak to the human dignity, the suffering, the hopes, the dreams, the lives gained, and the lives lost by the people we study’ (p. 15).^32^ Such a research frame enables researchers to raise participants’ awareness about socio-political issues that influence health outcomes and identify areas for social change.

## Data collection

We purposely recruited Black women aged 18 years and older who were pregnant or had given birth in the last 3 years, could communicate in English, and resided within the GTA. The recruitment of participants spanned over 4 months beginning June 2022 to October 2022. Recruitment fliers were distributed via social media, community-based organizations, and Black associations and groups. We also used snowball sampling by asking participants who were interviewed to refer to other women. We reached out to 28 women, and 24 consented to be interviewed, while the remaining 4 did not respond and no reasons were provided. From an onto-epistemological and methodological standpoint, a sample of 24 is deemed appropriate based on the aim of this study but also consistent within the discipline of qualitative inquiry. P.N.B. conducted a virtual semi-structured interview via Zoom, Google Meet, and over the telephone based on participants’ preference. To avoid leading participants, we chose not to ask them directly about obstetric racism, but instead, we invited them to share their experiences receiving care during pregnancy and childbirth. We critically engage participants to explore their understanding and the meanings they assigned to their encounters with healthcare providers using the following questions in addition to those listed on our interview guide: (a) please tell me about the nature and type of care you received during pregnancy or childbirth, (b) what do you think may have influenced the nature and type of care you received from healthcare providers? (c) please describe how the type of care you received made you feel and your decision to seek healthcare in future and (d) what do you think should be done to improve healthcare experiences of Black women during pregnancy and childbirth?

Although we had an interview guide, we took clues from the participants’ responses to rephrase and refine the questions during the interview. In furthering our understanding of participants’ responses to the interview guide, we used probes to clarify and critically explore what participants shared in depth. The interviews were conducted in English by the first author (P.N.B.). Each interview lasted between 30 and 60 minutes and were audio-recorded, and transcribed. All participants received a CAD$50 gift card each after the interview. Prior to the interview, we collected data on the sociodemographic information of the participants, including age, education, employment status and income. These findings are presented in Table 1.

**Table 1.** Participants’ sociodemographic characteristics.

Characteristics	N (%)
Age range (years)	
18–24	2 (8.3)
25–34	16 (66.7)
>35	6 (25)
Educational level	
High school diploma	1 (4.2)
College	4 (16.7)
Bachelor’s degree	15 (62.5)
Master’s degree	4 (16.7)
Employment status	
Employed	23 (95.8)
Unemployed	1 (4.2)
Individual income status (CAD$)	
21,000–30,000	1 (4.2)
31,000–40,000	3 (12.5)
41,000–50,000	4 (16.7)
>51,000	16 (66.7)

## Data analysis

A thematic analysis was used to generate a meaningful and compelling understanding of the data.^33^ We chose thematic analysis because of its flexibility, utility, and application to different range of epistemological and theoretical orientations.^34^ Both inductive and deductive techniques were used to generate a broader, comprehensive, and contextual interpretation of the entire data set. We began the data analysis by immersing ourselves in the data through repeated and actively listening to the recordings and reading of the transcripts. This process allowed us to gain insight into the ‘depth and breadth of the content’ (p. 16)^33^ of the data. Next, we documented our initial analytic impressions and generated codes using direct words or statements made by participants and concepts from the obstetric racism literature. Two research team members (P.N.B. and N.P.) independently coded the transcripts and met regularly to review and revise the codes to maintain consistency across the coding process. We subsequently sorted and grouped the codes into potential themes by comparing, merging, and constructing thematic maps. To ensure themes are closely linked and reflective of the data,^33^ we derived some of the themes based on words or statements expressed by the participants.

We named and defined the themes to capture the essence and meanings reflected in the participants’ experiences. The research team met to review and refine the themes and to select extracts from the data to construct a compelling accounts of the participants. We worked collaboratively as a team to identify areas of overlap and clearly define each theme’s scope and boundaries.^33^ All members of the research team met to discuss the best approach to construct a detailed, comprehensive, and compelling accounts taking into consideration our overarching research question. Through open dialogue, it was agreed that P.N.B., who has extensive experience in qualitative research, organized the findings. Other members (N.P., B.B., E.A.B., A-a.O. and S.S.) contributed to writing, reviewing, and editing the articles.

## Trustworthiness

We maintained trustworthiness by purposely sampling Black women who were pregnant or had given birth to ensure we were able to provide a thick description of participants’ experiences from different sociodemographic backgrounds. To increase the scope and adequacy of the data, we carefully listened to each audio to refine the next interview, and we continuously collected data until we reached saturation – that is, when no new information was being provided or found. We kept detailed notes of the analytic process, organized the findings into a compelling story, and used quotes from the diverse participants to illuminate each theme. B.B., E.A.B., S.S. and A-a.O. meticulously transcribed the data, while P.N.B. and N.P., with their expertise in qualitative research, reviewed the transcripts and independently coded the data. The research team frequently met to review, discuss and resolve disagreements through dialogue and finalize the themes. As Black women scholars and researchers, we were reflexive of our lived experiences and recognized how our positionality might impact on the research process and the data interpretation; hence, we wrote our introspection in a reflexive journal and discussed them openly during our frequent meetings. We also had two Black colleagues who provided a critical and independent review of the final draft to ensure we generated a compelling story grounded in the lived experiences and the voices of our participants. NVivo version 12 Release 1.7.1 was used to manage and organize the data analysis.

## Ethical consideration

The Toronto Metropolitan University Ethics Board approved the study (REB reference: 2022-207). All participants provided written informed consent before they were interviewed. The informed consent form included a brief description of the study aim, data collection procedure, and the risk and benefits. The interviews were conducted virtually, and participants were asked to turn off any cameras to ensure privacy and confidentiality. The interviews were recorded with a recording device and in line with ethical considerations governing this study; all audio recordings were immediately erased after transcription was completed and verified. Participants were informed during the interview to avoid mentioning names or institutions to prevent readers from linking the quote back to individuals. All transcripts were further reviewed to ensure identifying information, including names of healthcare providers and institutions, were removed or replaced with pseudonyms. Each transcript is assigned an alphanumeric code, which was retained during the data analysis and write-up.

## Findings

**Figure 1.**
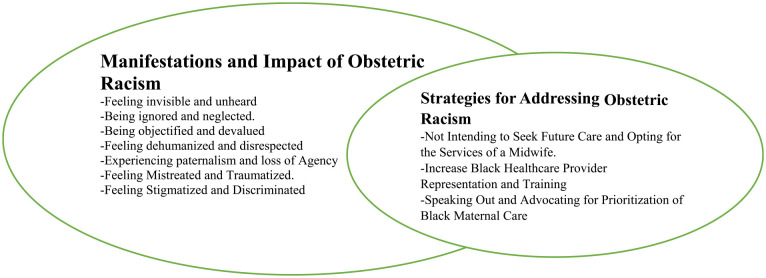
Themes and subthemes.

Table 1 above represents the demographic characteristics of participants. Two overarching themes were generated to illuminate the participants’ experiences: (1) Manifestations and Impact of Obstetric Racism and (2) Strategies for Addressing Obstetric Racism. Figure 1 below presents the themes and their subthemes.

## Manifestations and impacts of obstetric racism

Participants recounted their experiences of obstetric racism and the profound impact it had on the quality of perinatal care they receive. Many women shared their experiences of manifestations of obstetric racism including feeling invisible and unheard, being ignored and neglected, feeling objectified and devalued, feeling dehumanized and disrespected, experiencing paternalism and loss of agency, feeling mistreated and traumatized, and feeling stigmatized and discriminated.

## Feeling invisible and unheard

Feeling invisible and unheard was a common narrative threaded across Black women’s accounts. Participants described that despite standing in proximity to healthcare providers, their presence were unacknowledged. The women regarded the action of healthcare providers as intentional and being done on purpose. One participant shared an encounter with a healthcare provider in a birthing unit:

When I came into the birthing unit in labour, the nurse was just on the computer. I understand you need to do your work but at least acknowledge that I’m there because I’m going through stuff and you’re not acknowledging me. You’re doing it on purpose because you’re not acknowledging my presence when I have been standing there for five to six minutes. (BW8)

Another participant described how their invisibility resulted in their concerns being disregarded and considered incredible by healthcare providers:

I was explaining to her about labour pain, and no one was listening to me. I just felt like when I was explaining he wasn’t listening to me . . . it was annoying not being listened to and believed. It’s like I kind of just gave in and okay you’re the expert. As I look back, I’m like why didn’t you listen to me? (BW14)

Women also described how the experience of invisibility went beyond the confines of the hospitals to include other areas of the healthcare:

I was in the queue at the prenatal and I had to register to do a glucose tolerance test. When I was at the lab to do this test and I said hello to the technician, she was doing her work and did not acknowledge me meanwhile I was standing by her. I said hello I was here for a while, and she said oh sorry I didn’t see you and I’m like with all my Blackness you couldn’t see me? (BW15)

For most women, their encounters with the health system made them question how their Blackness was perceived and interpreted by the health providers. BW15 further described how the health system by design is set up to make them feel invisible and their voices go unheard when said: ‘If you’re a Black woman and accessing health services, it’s like you’re not seen. You can be there, but you are not seen at all, and you are not heard’. Several participants shared similar sentiments about ways the healthcare system reproduces and sustains their invisibility.

## Being ignored and neglected

Women shared their experiences of being ignored and neglected by health care providers at a time when they needed care. Participants described prejudice about Black women’s capacities to endure pain led to healthcare providers refusing to provide pain relief during labour. A woman recounted,

I was ignored because they see me and feel like I’m a strong Black woman and I can tolerate pain. For my first son, I waited an hour and a half for an epidural. But when I first went into the room, they asked if I wanted an epidural, and I was like yes. They’re like okay we’ll get you the doctor and the doctor came to my door and he’s like I’ll be right back and then never came back. (BW3)

Another participant also shared an encounter with a healthcare provider who ignored her despite experiencing severe morning sickness:

I was very sick. I’m always vomiting. I lost weight. I couldn’t gain weight in my pregnancy. I went to the doctor, and I was telling him about it and he [the doctor] just brushed it off and said you are going to get over it, it’s just morning sickness. But I have an extreme case of morning sickness, hyperemesis gravidarum and I was really dehydrated in my pregnancy and that should signal them that something is wrong. They just brushed that off and totally ignored all that. It is very disappointing and actually frustrating. (BW21)

Participants expressed their frustration about being dismissed by healthcare providers, contributing to their inability to receive urgent care and attention. Others also shared instances of being left unattended in a hallway for prolong hours:

They put me in a hallway for 15 hours on a gurney that they are running a blood test. I mean running those tests isn’t the issue. I just feel like you could have admitted me. You could have given me a meal. If I was a White woman, would they have left her in a hallway for 15 hours to fend for herself while pregnant? I think that they would have been a bit more mercy. (BW2)

Neglect, commonly reported and described in the accounts of participants as failure of healthcare providers to provide necessary care, was captured by other participants:

I was neglected. Nobody cared when I needed help. I don’t think I had the quality of care as a Black pregnant woman that a White person would have received. I feel like I was more of an obligation and than a main priority. (BW3)

Many described receiving less quality care than their White counterparts would have received. Neglectful actions contributed to women feeling their health and well-being were not a priority for healthcare providers.

## Being objectified and devalued

Black women felt healthcare providers objectified them and treated them as just numbers that needed to be processed through the healthcare system. Many described being made to feel unworthy and undeserving of care and attention. Several participants shared their experiences of how healthcare providers treated them as ‘things’ or ‘orders’ to be passed through the production line:

You’re seen as another thing coming through the door and what’s the quickest way to get you out the door to make room for other people that they feel are more worthy of their beds . . . So you’re almost being funneled through like an assembly line and only just lose your baby in the end or go through a really traumatic experience. (BW23)

It just felt like they see you as an order that they have to get through. Is like in order, to finish my shift I have to complete this order or project. That’s kind of how it felt to me . . .

It felt like I was just a process that she had to go through to get through her day kind of thing. (BW9)

Participants felt that being reduced to numbers denied them of their sense of personhood and resulted in indifference and lack of responsiveness from healthcare providers. Some women described how objectification contributed to the failure of healthcare providers to interact and communicate with them:

When I was in labour a physician just came in acting like I was just like a number, looking at the chart with no communication. It was just like you just come in, the baby’s out, you get sewn up. This physician when she came in you just felt like a number. (BW5)

Others felt that the manner in which healthcare providers related and treated them is a broader reflection of how the health system is set up to denigrate Black women:

When you look at how the health system treats Black women it makes me feel like I’m just a number, just get her in and get her out. . . . it’s like they just found another number and I am that number in the hospital bed and that’s literally how that situation felt. (BW18)

Another said, ‘This whole health system just sees pregnant Black women like you’re a statistic’ (BW23). For many women, the healthcare system perceived the bodies of Black women as data required for advancing the interest of physicians and healthcare institutions which contributed to dehumanizing them.

## Feeling dehumanized and disrespected

Dehumanization is reflected in the participants’ accounts of care that lacks dignity and compassion. For example, women recounted their experiences of feeling dehumanized and disrespected by healthcare providers while accessing care during pregnancy or childbirth:

I’ve been to a mechanic and the car was treated with more respect than I was treated during labour. The fact that they never bothered after the baby was born, never bothered checking in was very dehumanizing, very dehumanizing . . . at no point did she (the doctor) offer respect, there was no humanity. (BW2)

Some described health providers completely disregarded the humanity of Black women. For example, BW10, who had gone to the hospital in labour, shared her recollection of how she was made to ‘feel less than a person all the time’ and ‘being treated differently’. A similar experience was shared by BW23, who said, ‘generally, the doctors and nurses do not see you like you are a human being’. A participant also shared her experience of how the lack of respect for her body and humanity provokedintense distress:

Having a health care provider treat you like you’re a garbage and violate you in a way that is extremely triggering was just degrading. It was blatant disregard for yourself, respect and who you are as a person which for me is just dehumanizing. (BW2)

Black women recounted that these experiences resulted in care that was inhumane and lacked compassion. For example, three of the participants said ‘like there was no compassion or understanding or anything, there was no human element to her [OB] interaction with me’ (BW9), ‘it was definitely lacking just humaneness, it just feels like they have just lost the humaneness in care at the moment’ (BW4) and ‘it was like the response was very unprofessional and there was no empathy or care. No friendliness, very cold, not even looking at you in the face’ (BW23).

## Experiencing paternalism and loss of sense agency

Black women reported losing their agency to influence and make decisions about their care needs. Many shared how healthcare providers failed to provide them with information and options to enable them to make informed decisions. Some participants felt healthcare providers exhibited paternalistic attitudes by determining their treatment pathways. A participant described such actions by healthcare providers and how it amounts to ‘infantilization’ as follows:

The doctor was not giving me all options or laying it out for me to choose. They’re deciding for me. It’s like infantilization of grown Black women and not allowing us to make our own decisions. You’re not really giving us the options or laying out what options are available. My OB didn’t even explain the options to me, and it is like they determined what pathway to go. (BW23)

In the accounts of some women, they were denied the right to exercise agency or have control over their care and treatment:

It’s like you don’t have a choice through the experiences that I’ve had giving birth. And unfortunately, it is not me alone. A lot of Black women have that experience . . . especially those who went through the traditional OB way or the hospital that I have spoken to. They are not that open with your choice . . . the OB even nurses aren’t really open to certain processes that you go through in your pregnancy. It’s not as flexible for lack of better words. It’s just pretty much routine without providing you with options for you to choose. (BW7)

Some participants described feeling disempowered because of their lack of control and ability to influence their owncare. Many participants expressed their frustration of being compelled to comply with the decisions of healthcare providers without the opportunity to participate in decisions about their care: ‘With the whole birthing experience, I didn’t feel empowered. I felt that I had to comply with what they wanted. It was very frustrating . . . I felt very limited, and it was very frustrating and disempowering’ (BW18) and ‘Going to the hospital was disempowering, it was like I had no control over what I wanted. It was whatever the doctor thinks is best’ (BW9).

Several women reiterated the need for healthcare professionals to respect their autonomy and their right to have a choice and control over their reproductive health decisions. BW4 emphasized that healthcare providers must give ‘Black women the agency and autonomy to make good decisions and the control over their choice, and they should just respect their decisions’. Several other participants re-echoed these sentiments.

## Feeling mistreated and traumatized

Several participants described enduring mistreatment from healthcare providers during their perinatal journeys. Women described their experience as acts of violence perpetrated against them by people they trusted to care for them, which ultimately left their lives shattered. A participant recounted her experience going through childbirth:

Going through the birthing process from the beginning to the very end was a traumatic experience . . . I think it is a type of violence. I feel that is a type of violence. If you’re Black, you’re pregnant experiencing all these I think it is a form of violence. I think it’s all violence because even though my body wasn’t touched. I feel like my experience was violence and it is also crushing . . . it’s like you are existent but you are erased violently through their action . . . I guess when somebody violently kills someone you erase the person out of this world. For me it sorts of erased me from my life and that’s how I felt and it affected me . . . I would say the first three months I felt I was just doing as a robot first and I rarely breastfed my child. I honestly felt disconnected from the child because of the trauma and violence I experienced. (BW15)

Another participant also described her experience of being abused by healthcare providers:

Mentally I was messed up because of the way they treated me in the hospital, you know it was like abuse. And after even having my baby, I fell into depression. I didn’t want her. I felt like she was not more valuable than I was. So, I didn’t want her. (BW10)

The experiences of abuse, trauma and violence from healthcare providers impacted on these women’s mental health and ability to connect with their babies and transition into motherhood successfully. Many women felt childbirth placed them in a vulnerable position that made it difficult to advocate for themselves. This vulnerability was exacerbated by the fear of being neglected and treated with no care and compassion:

The trauma for me is like being neglected at a time when I’m like giving birth . . . It’s just really hard and it’s really traumatizing. The traumatizing moment for me is the fact of not being able to help myself and depending on other people that show that they don’t care about me. (BW3)

A heightened sense of fear and helplessness was pervasive in the accounts of several participants. A participant elaborated on her fear and feelings of helplessness when she said:

I felt like I was really at the mercy of this doctor which was also very scary. Because it’s like you don’t want to anger them. Like I felt being at the mercy of them [healthcare providers] like what my options were. I didn’t feel well enough to advocate for myself . . . It’s like one of those things where you really don’t know what your options are in the thick of everything . . . and you’re in survival mode. (BW2)

Participants expressed feeling powerless and being compelled to endure mistreatment from healthcare professionals. Such experiences left women feeling demoralized and defeated:

It’s really demoralizing because you experience poor treatment in so many other facets of life. So many other areas whether it’s in the education system, whether you are dealing with housing or dealing with a job. Even in healthcare and pregnancy and childbirth you have to deal with these microaggressions, and you deal with systemic racism while you’re in a most vulnerable state . . . It makes you feel very defeated. (BW23)

Black women described their disappointment of having to deal with everyday racism in healthcare encounters.

## Being stigmatized and discriminated

Widespread stereotypes about Black women were pervasive in participants’ encounters with healthcare providers. Participants reported that healthcare providers carry common societal assumptions about Black pregnant women being single and unmarried. Some women felt these stereotypes influence how they were treated by healthcare providers:

Because of the stigmas about Black women . . . like healthcare providers will talk down on because they assume you are a single Black mother. You’re a single mom and you’re Black so you’re going to have people coming at you with a completely different attitude, completely different lens, the way that you’re spoken to is different. I absolutely believe all this is based on the way that Black women are perceived. (BW5)

One participant shared her experience of how healthcare providers assumed she was unmarried:

When I was in the hospital my husband wasn’t with me and they would ask me ‘is your boyfriend coming?’ . . . So the assumptions that they make about you . . . is very disheartening that they make these assumptions which are incorrect and they don’t give you the benefit of the doubt by saying ‘when will your husband be coming and the immediate thing tat is your boyfriend coming’. It’s just part of the stereotype about Black women that they portray out there. (BW1)

Young Black women describe feeling stigmatized and judged by healthcare providers despite having their partners:

I’m young and Black that was something that she [the nurse] thought. Oh, there’s another young Black couple that are not married and giving birth. So, I feel like there’s a lot of stigma and judgment when they see young Black couples. (BW8)

Some women felt that healthcare providers also made assumptions about their children. BW7 elaborated on this when she said ‘they just feel like another problematic child being brought into this world that’s going to cause X, Y, and Z issues. So, it doesn’t matter how they are going to treat you’. These assumptions influenced how healthcare providers treated them. A participant also described how she was stigmatized when a healthcare provider assumed she was a child bride:

The obstetrician who attended to me made a comment like, are you one of those child brides from Africa? . . . Even though he had my health card and other records. Why would you make a comment like that just because I am Black . . . I don’t think that if someone is a White immigrant from the UK the obstetrician would ask them if you were a child bride. (BW15)

Black women felt the stigma and judgement by healthcare providers affected their mental health. Participants described how the internalization of the stigma resulted in feelings of guilt and an impending sense of failure despite being capable of parenting the unborn child:

Mentally these things they prey on you when you go through them. It makes you just feel like you’re already starting parenthood and feeling like you’ve already failed, that you’re not good enough, like you don’t have enough to offer, like you’re doing something wrong. (BW10)

## Strategies for Addressing Obstetric Racism

Women described different strategies for responding to the experience of obstetric racism. These strategies were reflected in the themes of Not Intending to Seek Future Care and Opting for Midwifery Care, Increase Black Healthcare Provider Representation and Training, and Speaking Out and Advocating for the Prioritization of Black Maternal Care.

## Not intending to seek future care and opting for the services of a midwife

Black women express their distrust of the healthcare system and their intent not to seek healthcare in their subsequent perinatal journey. Participants felt the healthcare system has failed to meet their needs and expectations. Some women described that the trauma of going through the healthcare system led to their decision to seek alternative care:

The Canadian system and again Western medicine I don’t really stick to because of the trauma of having to deal with that. So I have decided to rely on my own, more natural, naturopathic care because when it comes to health I feel like Canadian or Western medicine will fail you when you least expect . . . That’s why I decided I won’t go to the doctors for some help, but I will try to figure it out first. I will try to do as much as I can, and I look for their help only when I feel like I’ve exhausted all options, and nothing is working. (BW18)

Mistrust fuelled by the attitudes and actions of health care providers contributed to Black women distancing themselves from seeking obstetric care despite the risk. In addition, other participants voiced their dissatisfaction by avoiding particular hospitals despite not being guaranteed good care elsewhere:

I would absolutely not go back to him. I definitely said that before . . . I wouldn’t, I wouldn’t even recommend it to anybody. I’ll be honest I would not. (BW21)

I won’t go there, and I don’t advise anybody to go to that Camp Trail Hospital [real name is anonymized] to have a baby. If they want to have a baby, they go to a different place because like I had my third child in a different hospital based on that experience. (BW20)

While some women have indicated their intent not to seek care from specific healthcare facilities, others felt they would have to scrutinize the background of the healthcare provider they intend to visit to ensure they are able to meet their expectations:

I am going to be a lot more vigilant of the types of professionals that I work with because being dismissed by people is definitely going to continue to happen. But I think it’s also looking for professionals who are looking to find the solution as opposed to those that just look to dismiss. So it’s working with healthcare professionals that are women or specifically Black women or finding people who have a level of empathy of what I’m going through I think is key. (BW2)

Several Black women discussed opting to seek the services of midwives in their subsequent pregnancies. A participant who opted to have midwifery care after a previous difficult encounter in a hospital described how that experience influenced her decision to seek the services of midwives:

Going forward, like I always have a midwife because I feel like the midwife, they treat you with a little more respect . . . I felt empowered for the first time after my first pregnancy. Throughout pregnancy I felt empowered being told like it’s your body and you have to consent. (BW12)

Black women spoke highly of midwives for providing respectful and personalized care, which is contrary to what they experienced at the hospital. For many participants, midwives embody all that they expect and hope for:

With midwives it’s just a gentler approach. They appreciate or consider pretty much the desires and what people want. I just feel labour is like a sacred time and I need people in my circle that I actually have a relationship with . . . I wanted to have a team that respected my choice, a bit more receptive, a bit more open with your personal choice, and respect my choice. (BW7)

## Increase Black healthcare provider representation and training

Women recommended that increasing Black representation in perinatal care settings is critical to addressing obstetric racism in the health system. In addition, participants believe that having more Black healthcare providers integrated throughout the healthcare system will make a difference in how they are treated and cared for:

Ideally what I would love to see is more Black doctors and more Black nurses integrated into the system. We should get more Black nurses and Black doctors to come in so I guess that would be inclusivity which I think will change things. (BW21)

Several participants believed that having healthcare providers who share similar racial backgrounds as them will contribute to fostering respectful engagement and non-judgmental care:

Is about finding someone that looks like you and understands your situation. Having someone who looks like you and given the training and providing education would make a difference even just in the comfort level and then not feeling like there’s judgement. Having that self awareness as a person to be able to show up in healthcare spaces . . . challenging those stigmas that are associated with women. (BW5)

Participants also identified the need for equity, diversity and inclusion, and antiracism training for perinatal care providers to address the biases and deeply held assumptions about Black women to promote empathic engagement and responsive care:

I think that there needs to be more education, and training. I think there needs to be EDI, and antiracism training to address these biases. It needs to be training. And I feel it should be like a competency, and people need to go through to show that they are competent. (BW15)

I think that nurses and doctors should have some sort of training on how to emotionally support Black women. It just feels like they’re medically trained but I feel like the emotional part, the empathy and sympathy is lacking. I feel that they need to be trained on how to give Black women care. (BW12)

## Speaking out and advocating for prioritization of Black maternal care

Black women spoke about sharing their experiences to raise awareness and stimulate action and change. Participants saw that participating in the research was an opportunity to have their voices heard and advocate for systemic change as an initial step:

I think more Black women need to speak up and I feel like it has to start with more people speaking up about the situation and letting people know this is what’s happening because sometimes it’s not even just maternity it’s the whole system. So, I think more Black women need to speak up and people need to start advocating for themselves and advocating for others. (BW11)

Participants recognized the importance of documenting and exposing the negative encounters they experience in perinatal care. Women believe that collaborating with Black scholars was critical to facilitate the necessary changes required to promote Black maternal health:

I do hear from experiences from other Black women that are actually negative . . . people like you [researcher] should go out there and actually get these things documented and put out into the media or into the public. So I think they just need to do more research and I think it does take other Black advocates to get that message across a bigger platform or whatever and it takes people like you [researcher] that to get it out there will definitely bring change. (BW21)

Women also recognized the obvious lack of funding dedicated to addressing Black women’s health issues. Many recommend the need to prioritize and address the inequity in access to funding for Black maternal research:

There are all these things that don’t work in our favor for women. But a lot of money goes into things like prostate cancer, a lot of money goes into things that are White male dominated and less to Black women dominated issues like Black maternal health. So obviously having more research and designated research funding for Black women and maternal care. (BW24)

## Discussion

Our findings show that obstetric racism was widely pervasive within the healthcare system and manifested in the attitudes and actions of healthcare providers. Providing quality maternity care is critical to promoting healthcare utilization and preventing of maternal morbidity and mortality. Obstetric racism in perinatal care has been widely documented in the United States as a significant contributor to inequity in access to care and disparities in maternal health outcomes.^11,19^ In our study, Black women described feelings of invisibility, of not being heard, of being dismissed, ignored and neglected, resulting in the failure of healthcare providers to respond to their needs urgently and timely. Racial hierarchy and the need to dominate and control enslaved Black women’s reproductive lives have historically ‘led to differential practices, tasks and clinical decisions’ (pp. 58).^17^

These differential practices continue to manifest in the maternity care encounters of Black women, including neglect and denial of pain medication and other life-saving treatments. Yet, discourses about Black maternal morbidity and mortality primarily focused on the impact of stress and pathological risk factors, completely disregarding ways obstetric racism in the healthcare experiences of Black women profoundly influenced and shaped maternal health outcomes. Because obstetric complications may occur without warning, differential treatment may increase the risk of adverse maternal and newborn outcomes. While participants felt the lack of responsiveness of healthcare providers towards them was not surprising, it is also a broader reflection of the ways institutional and interpersonal racism within healthcare systems contribute to less-than-ideal maternity care and lack of prioritization of Black maternal healthcare issues.

Objectification of pregnant and birthing Black bodies was widely pervasive in the healthcare encounters of Black women. Our findings reveal that Black women’s bodies were treated as disposal objects with many recounting instances when they were treated as ‘a number’, ‘a statistic’ ‘a garbage’, ‘a product’ or ‘a project’. These findings are consistent with the dark history of medical experimentation when medical professionals show complete disregard of Black bodies and reducing them to clinical and experimental materials.^17,18^ When a person is viewed as ‘an object to be manipulated and controlled’ (p. 70),^35^ it sets the process for their dehumanization. Black women felt they were treated less than humans and less deserving of humane and respectful care, justifying the mistreatment from healthcare providers. The legacy of slavery continues to shape the delivery of medical care to Black people and how they are treated in healthcare encounters.^17^ Our findings revealed that objectification and dehumanization contributed to an overall lack of compassion, dignity and respectful care for Black women. These experiences contributed to feelings of abandonment, resentment and disillusion among Black women in this study who questioned whether their health and well-being were a priority to healthcare providers.

The unequal power relations between Black women and healthcare providers increase their risk and vulnerability to reproductive abuse and violence.^14^ Such vulnerability was commonly shared by most participants in this study who felt their parturient state coupled with medical dominance rendered them powerless to negotiate care and expose them to mistreatment and violence. Black women recounted experiences of violence receiving care during childbirth which left them horrified, obliterated and traumatized. Although women described not bodily harmed physically, the degrading and dehumanizing treatment they received were extremely triggering and traumatic. This experience left many Black women feeling unsafe and terrified in an environment where they are expected to be treated with compassion and care. Consistent with the mental health impacts of obstetric violence,^36^ participants felt disengaged and disconnected from their newborns, thereby threatening infant–parent attachment. The trauma of childbirth along with racism-related trauma from healthcare provider mistreatment can elicit a complex mental and emotional response that have far reaching consequences on the health and well-being of Black women and their infants. Risk of postpartum mood disorders among Black women has been attributed to stress and lack of support;^37^ however, our findings reveal that obstetric racism may increase the risk of postpartum depression and post-traumatic stress disorder among Black mothers. Given the significance of this finding, additional research is needed to examine the relationship between obstetric racism and posttraumatic stress disorder after childbirth among Black women.

Our findings also revealed that medical paternalism was pervasive, reminiscing the era of reproductive experimentation that stripped enslaved Black women of their right to autonomy and control.^18^ Paternalistic attitudes take different forms, including being presented with very limited choices^23,25^ or receiving inadequate health information from healthcare providers,^26^ both of which are consistent with the experiences shared by our participants. For example, participants felt infantilized and treated as incompetent and incapable of making healthcare decisions. These experiences deny them the right to exercise agency and control over their healthcare decisions. The experiences of Black women reflect ways institutionalized paternalism continues in covert and overt ways to lay claim to the reproductive life of Black women.^14^ This was reflected in the experience of participants who felt healthcare providers offered them with limited to no options and forced them to comply with whatever options were being presented.

Overall, these experiences compromised the therapeutic relationship between Black women and healthcare providers,^38^ further undermining their ability to engage with healthcare providers and to seek healthcare. There was also a heightened sense of fear associated with lack of safety and prioritization of perinatal care needs of Black women. This contributed to deepening mistrust of the healthcare system and future decision not to seek care. It is, therefore, imperative to address obstetric racism in Canadian healthcare system to avert looming maternal health crises like that of the United States. Midwives have been reported to be critical in addressing racial inequities and improving optimal birth outcomes for Black women.^39–41^ This understanding resonated in our participants’ narratives, who felt the philosophy of midwifery care aligns with their expectation of personalized and respectful care. Nearly all women had expressed intent to resort to the services of midwives in their subsequent pregnancy and childbirth journeys. Reproductive justice advocates have indicated that while the demand for midwifery care among Black women is increasing, the lack of culturally diverse midwifery workforce may deny many Black women access to these services.^40–43^

While this study addressed a significant gap in Black maternal health research in Canada, there are limitations. First, the findings are based on the experiences of Black women and does not capture the perspective of health care providers. Second, the research did not measure quality of care since findings are based on the perspectives of Black women. Nonetheless, this article is the first to document the experiences of obstetric racism among Black women in Canada. Future research should consider exploring the perspective of healthcare providers to identify opportunities for change.

## Conclusion

Our findings reveal ways obstetric racism impacts on the experiences and the quality of maternity care for Black women. Experiencing reproductive injustice and obstetric racism can unermine access to quality care and result in poor maternity outcomes. Providers’ presumptions and biases towards Black women reflected in the disrespectful and discriminatory treatment contributed to impacting on therapeutic relationship. We argue for the need to develop and implement anti-Black racism perinatal care models that considers how the history of reproductive injustice against Black women is reproduced and reinforced through acts of commission or omission. This model will support the training and delivery of culturally relevant, respectful and humanized perinatal care for Black women. There is the need for investment in Black maternal health research in Canada to generate meaningful evidence to inform policy and interventions to advance maternal health equity for Black women. To this end, we call on funding agencies to prioritize research funding opportunities to support Black maternal health research. This study focused on Black mothers’ experiences to highlight the issues and specific impacts on the quality of maternity care and to draw attention to the necessity of providing culturally appropriate care approaches to improve pregnancy outcomes for Black mothers and their babies.

## Supplemental Material

sj-docx-1-whe-10.1177_17455057231199651 – Supplemental material for Obstetric racism and perceived quality of maternity care in Canada: Voices of Black women Click here for additional data file. Supplemental material, sj-docx-1-whe-10.1177_17455057231199651 for Obstetric racism and perceived quality of maternity care in Canada: Voices of Black women by Priscilla N Boakye, Nadia Prendergast, Bahareh Bandari, Eugenia Anane Brown, Awura-ama Odutayo and Sharon Salami in Women’s Health

sj-docx-2-whe-10.1177_17455057231199651 – Supplemental material for Obstetric racism and perceived quality of maternity care in Canada: Voices of Black women Click here for additional data file. Supplemental material, sj-docx-2-whe-10.1177_17455057231199651 for Obstetric racism and perceived quality of maternity care in Canada: Voices of Black women by Priscilla N Boakye, Nadia Prendergast, Bahareh Bandari, Eugenia Anane Brown, Awura-ama Odutayo and Sharon Salami in Women’s Health

## Declarations

**Ethics approval and consent to participate:** The research and study materials were reviewed and approved by Toronto Metropolitan University Research Ethics Board (REB reference: 2022-207). The data collection and conduct of the research was in accordance with the principles governing ethical conduct of research. Participants received a copy of the consent form a week prior to the interview, and all provided a written informed consent.

**Consent for publication:** Not applicable.

**Availability of data and materials:** The data (transcripts) are not available publicly as participants did not provide consent for data sharing or for it to be made available to others. All requests should be referred to the first author (P.N.B.). The quotes used in this publication are subject to the consent agreement signed by the participants and consistent with Toronto Metropolitan University Research Ethics Board.
