# Supplementary material for: Obstetric racism and perceived quality of maternity care in Canada: Voices of Black women
Source: Womens Health (Lond). 2023 Sep 29;19:17455057231199651. doi: 10.1177/17455057231199651 (PMC10542226; doi:10.1177/17455057231199651)
Supplement: sj-docx-1-whe-10.1177_17455057231199651 – Supplemental material for Obstetric racism and perceived quality of maternity care in Canada: Voices of Black women [file sj-docx-1-whe-10.1177_17455057231199651.docx]

Manuscript: Obstetric Racism and Perceived Quality of Maternity Care in Canada: Voices of Black Women

Consolidated criteria for reporting qualitative studies (COREQ): 32-item checklist.

| **Topic** | **Item No.** | **Guide questions/description** | **Reported on Page #** |
| --- | --- | --- | --- |
| **Domain 1: Research team and reﬂexivity** | | | |
| Personal Characteristics | | |  |
| Interviewer/facilitator | 1 | Which author/s conducted the interview or focus group? | Page 4 |
| Credentials | 2 | What were the researcher’s credentials? E.g. PhD, MD | Title page |
| Occupation | 3 | What was their occupation at the time of the study? | Title page |
| Gender | 4 | Was the researcher male or female? | Page 7 |
| Experience and training | 5 | What experience or training did the researcher have? | Page 7 |
| Relationship with participants | | | |
| Relationship established | 6 | Was a relationship established prior to study commencement? | No. Not reported on page |
| Participant knowledge of the interviewer | 7 | What did the participants know about the researcher? e.g. personal goals, reasons for doing the research | Page 7 |
| Interviewer characteristics | 8 | What characteristics were reported about the inter viewer/facilitator? e.g. Bias, assumptions, reasons and interests in the research topic | Page 7 |

| Domain 2: study design | | | |
| --- | --- | --- | --- |
| Theoretical framework | | | |
| Methodological orientation and Theory | 9 | What methodological orientation was stated to underpin the study? e.g. grounded theory, discourse analysis, ethnography, phenomenology, content analysis | Page 4 |
| Participant selection | | | |
| Sampling | 10 | How were participants selected? e.g. purposive, convenience, consecutive, snowball | Page 4 |
| Method of approach | 11 | How were participants approached? e.g. face-to-face, telephone, mail, email | Page 4 |
| Sample size | 12 | How many participants were in the study? | Page 4 |
| Non-participation | 13 | How many people refused to participate or dropped out? Reasons? | Page 4 |
| Setting | | | |
| Setting of data collection | 14 | Where was the data collected? e.g. home, clinic, workplace | Page 4 |
| Presence of non-participants | 15 | Was anyone else present besides the participants and researchers? | None reported |
| Description of sample | 16 | What are the important characteristics of the sample? e.g. demographic data, date | Page 8 |
| Data collection | | | |
| Interview guide | 17 | Were questions, prompts, guides provided by the authors? Was it pilot tested? | Page 4-5  Interview guide |
| Repeat interviews | 18 | Were repeat interviews carried out? If yes, how many? | None |
| Audio/visual recording | 19 | Did the research use audio or visual recording to collect the data? | Page 5 |
| Field notes | 20 | Were ﬁeld notes made during and/or after the interview or focus group? | None |
| Duration | 21 | What was the duration of the interviews or focus group? | Page 5 |
| Data saturation | 22 | Was data saturation discussed? | No |
| Transcripts returned | 23 | Were transcripts returned to participants for comment and/or correction? | No |
| **Domain 3: analysis and ﬁndings** | | | |
| **Domain analysis** | | | |
| Number of data coders | 24 | How many data coders coded the data? | Page 8 |
| Description of the coding tree | 25 | Did authors provide a description of the coding tree? | 5 -6 |
| Derivation of themes | 26 | Were themes identiﬁed in advance or derived from the data? | Page 6 |
| Software | 27 | What software, if applicable, was used to manage the data? | Page 7 |
| Participant checking | 28 | Did participants provide feedback on the ﬁndings? | No |
| Reporting | | | |
| Quotations presented | 29 | Were participant quotations presented to illustrate the themes/ﬁndings? Was each quotation identiﬁed? e.g. participant number | Page 9-20 |
| Data and ﬁndings consistent | 30 | Was there consistency between the data presented and the ﬁndings? | Page 9-20 |
| Clarity of major themes | 31 | Were major themes clearly presented in the ﬁndings? | Page 9-20 |
| Clarity of minor themes | 32 | Is there a description of diverse cases or discussion of minor themes? | Page 9-20 |

Developed from: Tong A, Sainsbury P, Craig J. Consolidated criteria for reporting qualitative research (COREQ): a 32-item checklist for interviews and focus groups. International Journal for Quality in Health Care. 2007. Volume 19, Number 6: pp. 349 – 357
