# Supplementary material for: Obstetric racism and perceived quality of maternity care in Canada: Voices of Black women
Source: Womens Health (Lond). 2023 Sep 29;19:17455057231199651. doi: 10.1177/17455057231199651 (PMC10542226; doi:10.1177/17455057231199651)
Supplement: sj-docx-2-whe-10.1177_17455057231199651 – Supplemental material for Obstetric racism and perceived quality of maternity care in Canada: Voices of Black women [file sj-docx-2-whe-10.1177_17455057231199651.docx]

**Interview guide**

Hello, my name is Priscilla Boakye, and I would like to welcome you and thank you for your interest in participating in this study. In this interview, I will be asking questions about your experience seeking health during pregnancy and or childbirth as a Black woman. I will be recording the interview to ensure I adequately capture your story. All electronic and hard copy of the information you provide will be kept securely. The information you will be providing will be shared with members of the research team only with all your identifiable information removed. You may choose not to share any information you might be uncomfortable with at any time. You can also withdraw your consent to participate at any time.  Please do you have any questions?

1. Please tell me about the nature and type of care you receive during pregnancy or childbirth.
2. What do you think may have influenced the nature and type of care you received from the healthcare providers?
3. Please describe how the type of care you received made you feel and your decision to seek healthcare in future.
4. What perception do healthcare providers have about Black women pregnancy and childbirth?
5. How have these perceptions of healthcare providers about Black women’s pregnancy and childbirth shaped the kind of care you receive?
6. How were your health concerns perceived by the health care provider?
7. What do you think may have influenced the way the healthcare provider responded to your concerns and how did that make you feel?
8. Did you have control over your health decisions, and did you feel supported by the health care provider?
9. How were you able to negotiate/navigate through the health system to get the needed care?
10. How has your overall experience influenced your future decision to seek health care and choice of future healthcare provider?
11. What do you think should be done to improve health care experiences of Black women during pregnancy and childbirth?
